# Peritoneal chemotherapy delivery systems for ovarian cancer treatment: systematic review of animal models

**DOI:** 10.3389/fonc.2024.1487376

**Published:** 2025-01-08

**Authors:** Marcelo Simonsen, Rossana Verónica Mendoza López, Simone Maistro, Lucas Takeshi Ikeoka, Glaucia Fernanda de Lima Pereira, Ademar Benévolo Lugão, José Carlos Sadalla, Maria Lúcia Hirata Katayama, Maria Aparecida Azevedo Koike Folgueira

**Affiliations:** ^1^ Departamento de Radiologia e Oncologia, Instituto do Cancer do Estado de Sao Paulo, Hospital das Clinicas, Faculdade de Medicina, Universidade de Sao Paulo (HCFMUSP), Sao Paulo, SP, Brazil; ^2^ Gynecology and Obstetrics Department, Instituto de Assistência Médica ao Servidor Público Estadual (IAMSPE), São Paulo, SP, Brazil; ^3^ Departamento de Radiologia e Oncologia, Comprehensive Center for Precision Oncology (C2PO), Centro de Investigação Translacional em Oncologia (CTO), Instituto do Cancer do Estado de Sao Paulo, Hospital das Clinicas, Faculdade de Medicina, Universidade de Sao Paulo (HCFMUSP), Sao Paulo, SP, Brazil; ^4^ Faculdade de Medicina, Undergraduate program, Universidade de Sao Paulo (FMUSP), Sao Paulo, SP, Brazil; ^5^ Instituto do Cancer do Estado de Sao Paulo, Hospital das Clinicas, Faculdade de Medicina, Universidade de Sao Paulo (HCFMUSP), Sao Paulo, SP, Brazil; ^6^ Nuclear and Energy Research Institute, IPEN-Comissão Nacional de Energia Nuclear (CNEN)/SP—University of São Paulo, São Paulo, SP, Brazil; ^7^ Departamento de Ginecologia e Obstetrícia, Instituto do Câncer do Estado de Sao Paulo do Hospital das Clínicas da Faculdade de Medicina da Universidade de São Paulo, Sao Paulo, SP, Brazil

**Keywords:** ovarian cancer, drug delivery systems, animal model, intraperitoneal chemotherapy, meta-analysis

## Abstract

**Introduction:**

Intraperitoneal chemotherapy for ovarian cancer treatment has controversial benefits as most methodologies are associated with significant morbidity. We carried out a systematic review to compare tumor response, measured by tumor weight and volume, between intraperitoneal chemotherapy delivered via drug delivery systems (DDSs) and free intraperitoneal chemotherapy in animal models of ovarian cancer. The secondary aim was to assess the toxicity of DDS-delivered chemotherapy, based on changes in animal body weight.

**Methods:**

Based on PRISMA and SYRCLE guidelines, we identified 38 studies for review, of which 20, were used in the meta-analysis. We evaluated outcome, through tumor volume and tumor weight and, toxicity, through animal weight. Analysis was based on drugs employed and treatment duration.

**Results:**

Most studies were performed on mice. Ovarian cancer cell lines most commonly used to induce xenografts were SKOV3 (19 studies) and A2780 (6 studies). Intraperitoneal device, also known as drug delivery systems (DDS), consisted in nanoparticles, hydrogels, lipid polymer and others. The most commonly used drugs were paclitaxel and cisplatin. Most studies used as the control treatment the same chemotherapy applied free intraperitoneally and tumor response/animal weight were evaluated weekly. There was a small benefit in overall tumor reduction in animals treated with intraperitoneal chemotherapy applied through the slow release device compared with animals treated with intraperitoneal free chemotherapy, as evaluated through tumor weight - results in standardized mean difference. (-1.06; 95% CI: -1.34, -0.78) and tumor volume (-3.72; 95% CI: -4.47, -2.97), a benefit that was seen in most weekly evaluations and for most chemotherapy drugs, such as carboplatin (tumor weight: -5.60; 95% CI: -7.83, -3.37), paclitaxel (tumor weight: -1.18; 95% CI: -1.52, -0.83), and cisplatin (tumor volume: -2.85; 95% CI: -3.66, -2.04) carboplatin (tumor volume: -12.71; 95% CI: -17.35, -8.07); cisplatin (tumor volume: -7.76; 95% CI: -9.88, -5.65); paclitaxel (tumor volume: -2.85; 95% CI: -3.66, -2.04). Regarding animal weight, there was no weight reduction in animals treated with intraperitoneal chemotherapy applied through the slow-release device compared with animals treated with intraperitoneal free chemotherapy. However, significant heterogeneity was observed in some comparisons.

**Conclusion:**

slow-release devices are overall safe and effective in animal models of ovarian cancer. It was not possible to evaluate which one is the most promising device to treat ovarian cancer, because many different types were used to apply chemotherapy intraperitoneally.

**Systematic Review Registration:**

https://www.crd.york.ac.uk/prospero/, identifier CRD42021224573.

## 1 Introduction

Ovarian cancer is the most lethal gynecologic cancer, with the majority of cases being diagnosed when the patient already presents with ascites and peritoneal dissemination of the tumor, restricting the 5-year survival to 30% (1). This situation impairs complete cytoreduction, and limits the chemotherapy response (2–4). The majority of patients have relapsed disease (1, 5, 6).

The most common chemotherapy protocol for high grade advanced stage serous ovarian cancer is based on six cycles of systemic carboplatin and paclitaxel (1, 5). Toxicity is related to unfavorable drug distribution and results in frequent peripheral neuropathy, anemia, neutropenia due to bone marrow suppression, alopecia and gastrointestinal symptoms.

Peritoneal administration of chemotherapy has been implemented over the last few decades to increase drug concentration in neoplastic spots with acceptable systemic toxicity due to lower absorption (3, 7), but sequential outpatient administration did not improve survival in the most expressive clinical trial (8). The single application during cytoreduction of hyperthermic intraperitoneal chemotherapy (HIPEC) was effective in three clinical trials.

The rational of HIPEC is to directly or indirectly affect the residual neoplastic cells after cytoreduction. In this case, the desperitonized region, which is more prone to the adhesion of neoplastic cells, is protected by direct contact with the chemotherapy agent (9). The technique presents promising results even in patients with platinum resistance (10, 11). Studies show better survival compared with standard treatment, when performed during interval cytoreduction surgery with cisplatin (12) and in the context of secondary cytoreduction with cisplatin (13) or cisplatin associated with paclitaxel (10).

The main disadvantage of HIPEC is the higher surgical morbidity and mortality. The most frequent complications are bleeding, surgical wound infection, sepsis, abscess, fistulas, renal failure, pleural effusion and hematological toxicity associated with chemotherapy (7).

Although some series present good results regarding the morbidity of the procedure (10), others report morbidity rates that exceed 50% of patients (14), encouraging studies in murine models for less toxic formulations.

The PIPAC (pressurized intraperitoneal aerosol chemotherapy) resembles the principles of HIPEC with an innovative technology for delivering drugs into the peritoneal cavity, that involves aerosolized chemotherapy delivered under pressure. Unfortunately, it is mainly offered to palliative patients, as there are still no randomized clinical trials evaluating PIPAC as first therapeutic option (15).

Chemotherapy slow-release devices, also known as drug delivery systems (DDSs), are designed to converge the advantage of *in loco* peritoneal treatment with lower toxicity. The classification of DDSs is based on its main mechanism of action and the types most currently used are liposomes, micelles and nanoparticles (16). Its efficacy in disease control has been demonstrated in several animal models but (17, 18) its application in humans is limited to a few studies on slow-release systems containing paclitaxel (19, 20). Several DDS formulations have been tested *in vitro* and in murine models, and most studies have been conducted in mice with ovarian tumor xenografts (17). The efficacy of various DDS formulations reinforces this therapeutic option and supports the creation of a device to be tested in animals with a larger peritoneal cavity.

The primary aim of this study was to compare the tumor response, assessed by tumor weight and volume, between intraperitoneal chemotherapy delivered via drug delivery systems (DDSs) and free intraperitoneal chemotherapy in animal models of ovarian cancer. The secondary aim was to evaluate the toxicity of intraperitoneal chemotherapy delivered through DDSs, compared to free intraperitoneal chemotherapy, based on changes in animal body weight.

## 2 Methods

The research protocol was inserted into the PROSPERO (Prospective Register of Systematic Reviews) platform with the register code CRD42021224573, following the PRISMA (Preferred Reporting Items for Systematic Review and Meta-Analysis) checklist recommendations (21). We performed a literature search using descriptors according to PICO methodology, as described in **Supplementary Table 1**: (Ovarian OR carcinomatosis) AND (Polymers OR Drug Delivery Systems OR Absorbable Implants OR Phospholipids OR Delayed-Action Preparations OR Infusion Pumps, Implantable OR Chitosan OR Polyvinyl Alcohol OR sustained release OR slow release OR controlled release OR membrane OR hydrogel OR Polyethylene Glycol Acid OR Implant System OR Injectable Biomaterial OR Continuous Release OR continuous intraperitoneal delivery OR continuous chemotherapy OR continuous docetaxel OR continuous cisplatin OR continuous paclitaxel OR continuous carboplatin OR micellar OR micelle) AND (Intraperitoneal OR peritoneal) AND (Toxicity OR survival OR treatment OR tumor burden). The search strategy focused on DDSs capable of slow-release chemotherapy in ovarian cancer animal models.

The literature search was performed on PubMed, MEDLINE, Embase, Cochrane Central Register of Clinical Trials and Web of Science, without any date restrictions on 2^th^ October 2023. All results were inserted into the Rayyan App, a multitask program created to enable better management and selection of papers (22). Repeated studies on different platforms were excluded, and papers were selected by six researchers working in blinded pairs. After interrupting the blind approach, discordant papers between the authors were reviewed by a third author for final judgment on inclusion.

The selection criteria included studies with peritoneal application of the device in which one or more of the following chemotherapy drugs were used: cisplatin, docetaxel, paclitaxel and carboplatin. The device type was restricted to gels, membranes, microdevices or micelles.

Study inclusion required at least one of the following outcomes: side effects (animal weight as a sign of toxicity), tumor response (assessed by tumor weight, volume or bioluminescence), or animal survival. Experimental group had be compared at least with one control group (no treatment, empty device or free chemotherapy) and minimal sample size and animal species were not an exclusion criteria. Only studies published in English were included. Studies on human beings were excluded. Data compilation for meta-analysis was prepared when at least three studies with the same outcome, unit of measure and standard deviation were present.

Authors of abstracts and posters were contacted digitally (email/LinkedIn platforms) and kindly asked to send additional data that would facilitate the inclusion of the research in the review.

The selected studies had their data extracted and inserted into a standardized Excel table by all researchers. The corresponding pair reviewed all data entries; text, tables or graphs had their numbers copied. When the results were presented only graphically, the *WebPlotDigitizer* program was used to identify the results more accurately. Data extracted from the papers consisted of authors, year of publication, number of animals in each group (experimental and control), cell lines, intervention, and outcomes.

Meta-analysis was performed using STATA MP version 14 software. When the variation in mouse weight was recorded as a percentage, we considered the initial weight to be 20g to include the data in the analysis.

We conducted a meta-analysis using a fixed-effect model. The outcome measures were assessed based on continuous variables. The effect of the treatment interventions and controls was evaluated by calculating mean differences and their corresponding 95% confidence intervals. The overall treatment effect was further assessed using the standardized mean difference (SMD). A p-value of < 0.05 was considered statistically significant. The degree of heterogeneity across studies was evaluated using the I² statistic, with the following interpretation: 0–25% indicating low heterogeneity, 26–50% moderate heterogeneity, 51–75% substantial heterogeneity, and >75% high heterogeneity (23).

### 2.1 Studies quality

The SYRCLE (Systematic Review Centre for Laboratory animal Experimentation) risk of Bias Tool was used to quantify the quality of the studies (24); this is an adaptation of the Cochrane Instrument developed specifically for animal studies. Selection, performance, detection, attrition and reporting biases were evaluated by the same researchers who selected the articles, in pairs.

## 3 Results

The search was carried out on October 2, 2023 and 399 studies were found in PubMed, 417 in Embase, 226 in Web of Science, 443 in the Virtual Health Library, 195 in Scopus and 23 in Cochrane, totaling 1,703 articles. It should be noted that the terms were searched in the title, abstract or entire text; only in Scopus was the search for terms restricted to the title and abstract, as the high number of articles in the text would make the selection of articles unfeasible.

**Figure 1:**
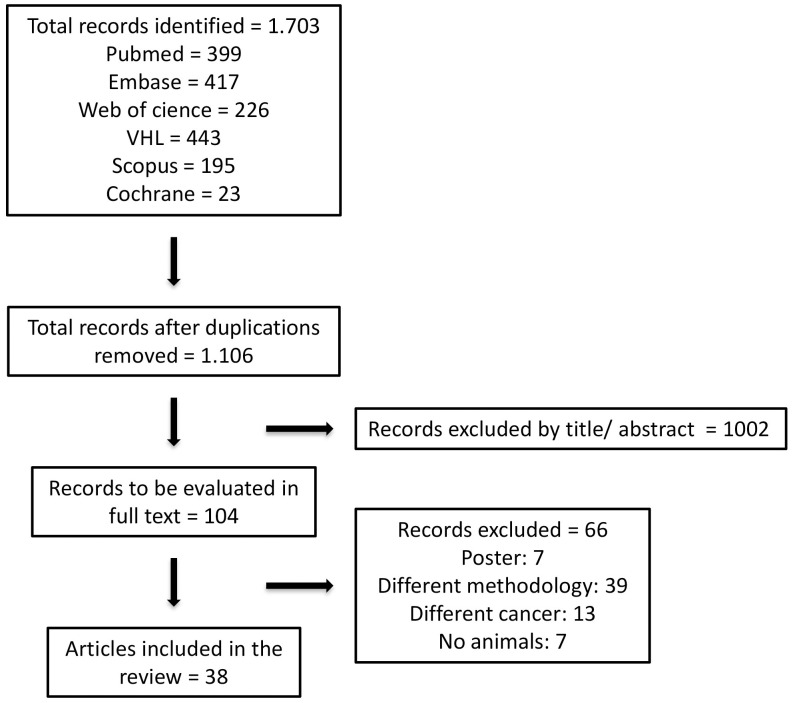
Flowchart of selected studies.

A total of 597 articles were excluded, resulting in an evaluation of 1,106 articles. Of these, 104 papers were selected based on the relevance of the title or summary for the final evaluation of the full text (**Figure 1**). It was not possible to get in contact with the authors of the six posters initially included.

### 3.1 Systematic review

A total of 38 articles fulfilled the inclusion criteria and were selected for a systematic review. The analysis was performed according to treatment duration and chemotherapy drugs used. A total of 11 studies published from databank inception up to 2010 were selected, from 2011 to 2015, 14 studies, from 2016 to 2023, 10 studies, and from 2021 to 2023, 3 studies.

Eighteen studies were excluded from the meta-analysis. The main reason for this was that the outcomes did not include the predefined parameters. The reasons for excluding these studies and their outcomes are summarized in **Table 1**.

**Table 1:** Main features of the studies selected for the systematic review and reason for exclusion.

Author	Year	Chemo	Physical Presentation	*N* Control *vs*. Experimental	Evaluated Outcomes	Exclusion Reason
Amoozgar et al. (53)	2014	paclitaxel	nanoparticles	16 *vs*. 8	survival	missing standard deviation
Bajaj et al. (28)	2012	paclitaxel	hydrogel	27 *vs*. 19	tumor weight	*
Bortot et al. (54)	2020	cisplatin	nanoparticles	12 *vs*. 7	bioluminescence and animal weight	*
Cho H and Kwon GS et al. (55)	2014	paclitaxel	micels and gel	10 *vs*. 10	bioluminescence and survival	missing standard deviation
Cho H and Lai et al. (56)	2013	paclitaxel	Micels	8 *vs*. 16	animal weight	*
Cho, S. and Sun et al. (45)	2015	cisplatin	nanoparticles	30 *vs*. 20	bioluminescence and tumor weight	*
Clercq et al. (27)	2019	paclitaxel	nanoparticles	22 *vs*. 44	animal weight	time treatment missing
De Souza R and Zahedi P et al. (33)	2010	docetaxel	hydrogel	12 *vs*. 36	bioluminescence	outcomes missing
Desale SS et al. (35)	2015	cisplatin and paclitaxel	nanoparticles	32 *vs*. 16	bioluminescence	outcomes missing
Gilmore D et al. (57)	2013	paclitaxel	nanoparticles	13 *vs*. 11	animal and tumor weight	*
Hagiwara et al. (58)	1993	cisplatin	microspheres	240 *vs*. 20	survival	outcomes missing
Chunbai He et al. (59)	2016	cisplatin	siRNA polymer	18 *vs*. 24	animal weight, tumor volume and survival	*
Ho et al. (60)	2007	paclitaxel	nanoparticles	6 *vs*. 3	–	outcomes missing
Kumagai et al. (25)	1996	cisplatin	microspheres	5 *vs*. 40	survival	outcomes missing
Lee, S.E. and Bairstow et al. (61)	2014	paclitaxel	nanoparticles	30 *vs*. 10	survival	outcomes missing
Li SD and Howell et al. (62)	2010	cisplatin	microparticles	14 *vs*. 6	survival	missing standard deviation
Lu H and Li B et al. (63)	2007	paclitaxel	nanoparticles	45 *vs*. 15	tumoral weight	*
Lu Z and Tsai M et al. (31)	2008	paclitaxel	microparticles	43 *vs*. 26	survival	outcomes missing
Padmakumar S et al. (64)	2019	paclitaxel	nanotextile	10 *vs*. 10	animal weight	*
Poon, C. et al. (43)	2016	carboplatin	nanoparticles	20 *vs*. 10	tumor weight and volume	*
Sun et al. (65)	2016	paclitaxel	nanocristals	27 *vs*. 18	bioluminescence and survival	missing standard deviation
Tong et al. (66)	2014	paclitaxel	liposome with nanoparticles	30 *vs*. 20	survival	missing standard deviation
Vassileva et al. (38)	2008	paclitaxel	device not specified	24 *vs*. 12	tumor weight	*
Wang et al. (47)	2020	cisplatin	polymer stent	12 *vs*. 6	tumor and animal weight	*
Xiao et al. (29)	2009	paclitaxel	nanoparticles	15 *vs*. 10	bioluminescence, tumor weight, animal weight and survival	*
Xie et al. (67)	2019	paclitaxel	microspheres	19 *vs*. 23	bioluminescence and Survival	missing standard deviation
Xiong et al. (68)	2012	paclitaxel	nanoparticles	6		no DDs with Slow release
Xu et al. (44)	2016	paclitaxel	hydrogel	24 *vs*. 8	tumor and animal weight	*
Yang et al. (69)	2017	gencitabin and paclitaxel	polymer	20 *vs*. 15	tumor and animal weight	*
Yang et al. (34)	2014	paclitaxel	microsphere	15 *vs*. 27	bioluminescence and PCI	missing standard deviation
Ye et al. (37)	2015	cisplatin	microdevice	40 *vs*. 26	bioluminescence, tumor and animal weight	*
Ye et al. (70)	2013	paclitaxel	liposome	27 *vs*. 21	tumor weight	missing standard deviation
Zahedi et al. (36)	2010	docetaxel and cepharanthine	hydrogel	12 *vs*. 6	tumor weight	*
Zahedi et al. (26)	2012	docetaxel	hydrogel	36 *vs*. 24	tumor volume	*
Zahedi et al. (32)	2009	docetaxel	hydrogel	4 x 8	tumor volume	missing standard deviation
Zhang et al. (71)	2022	docetaxel	micels	24 x 18	tumor and animal weight	*
Yamaguchi et al. (46)	2022	cisplatin	hydrogel	18 x 12	tumor volume and animal weight	*
Zhao et al. (72)	2022	cisplatin	nanotubes	15 x 5	animal weight	*

*Included in the meta-analysis.

Almost all studies were performed on mice, except for one performed on rats (25), and the experimental number varied from 3 (26) to 44 (27) animals, aged between 4 (28) and 12 (29) weeks. The unique study in porcine model was excluded in the initial phase due to lack information about DDS (30). The slow-release chemotherapy device was applied from 1 (27) to 28 (31) days after the induction of tumor formation. The most commonly used cell lines to induce xenografts were the ovarian cancer lineages SKOV3, in 19 studies and A2780, in six studies.

The models of slow-release chemotherapy devices used in each study are listed in **Table 1**. The device formulation showed significant variations among the studies. The most commonly used presentations consisted of nanoparticles (12 studies) and hydrogels (seven studies). The only standardized material used in more than one study (a total of three articles) was a lipid-polymer containing docetaxel (polygel), which showed good performance and low toxicity (26, 32, 33). The most commonly used chemotherapy drugs were paclitaxel (22 studies) and cisplatin (11 studies).

Most studies used only one chemotherapy drug but, Yang et al. (34), Desale et al. (35) and Zahedi et al. (36) used a combination of chemotherapy drugs. In addition Bajaj et al. (28) tested only one drug, paclitaxel, dissolved in Cremophor or DMSO, and assessed the outcomes separately.

Mouse survival was the most studied outcome, assessed in 19 studies. However, we did not evaluate this parameter because most studies presented the Kaplan Meier curve without confidence intervals. Tumor response was assessed based on tumor weight (17 studies). Xenograft bioluminescence was observed in 12 studies, and tumor volume in 5 studies. Mouse weights were quantified in 14 studies. In total, 18 studies compared outcomes of DDS vs intraperitoneal PBS, 10 of DDSs vs empty device (no chemotherapy), and 18 of DDS vs intraperitoneal free chemotherapy. We present results of comparisons between DDS versus intraperitoneal free in the text, and the other two DDS comparisons (with intraperitoneal PBS or DDS without chemotherapy) in **Supplementary Material**.

Most studies used as the control treatment the same chemotherapy, administered intraperitoneally in bolus, using various regimens, such as 1 dose every 3 days for 5 doses or 2 doses separated by 1 week interval, and others. The studies also compared DDS with the absence of treatment (with phosphate-buffered saline (PBS) infusion or application of a slow-release device without any drug).

**Figure 2:**
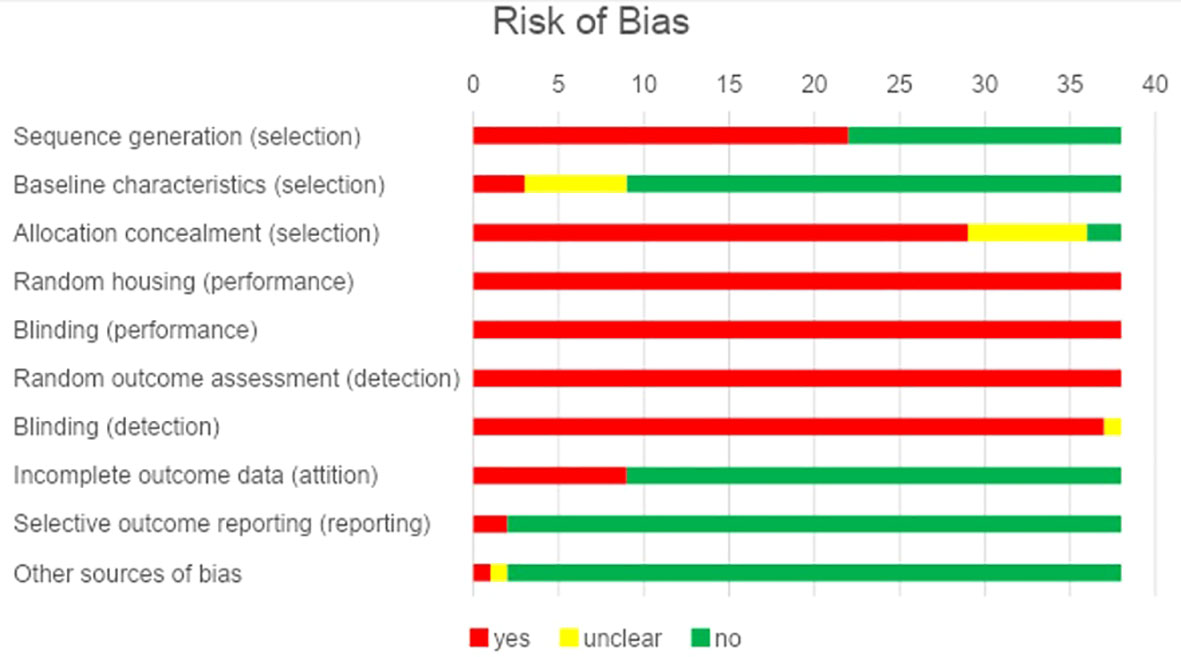
Risk of bias represented by the percentage of studies included.

The risks of bias are shown in **Figure 2**. Most studies have clearly described the methods used to equalize different groups under the same care. The random/alternating cage distribution in the vivarium or the blinding of caregivers was not reported in any study, negatively affecting the performance of the selection, performance and detection domains. The presentation of all data proposed initially in the methodology section, in the results section and the presentation of different parameters to quantify the tumor response (such as tumor weight and bioluminescence) guaranteed satisfactory results in attrition and selection bias.

### 3.2 Meta-analysis

Data selection for the meta-analysis was based on the outcomes of tumor volume, tumor weight, mouse weight and mouse survival in 6, 11, 16 and 19 studies, respectively. The large variation in the data units among the studies evaluating tumor growth by bioluminescence precluded their inclusion in the meta-analysis, even though it was described in 12 studies. Survival was not evaluated because most studies provided Kaplan Meier curve without Confidence intervals, which precluded meta-analysis.

In the meta-analysis, we evaluated the outcomes of each study considering weeks after the application of the slow-release device. We compared the results of the device with those of three control groups: I, peritoneal or intravenous application of the same free chemotherapy (without conjugated formulation and without release device), II, no treatment or PBS, and III, application of the slow-release device without chemotherapy, The first, presented in the text (II and III presented in **Supplementary Material**).

For the analysis of tumor weight and volume, animals were euthanized at different time intervals, and separate groups were analyzed at each time point. This approach allowed us to stratify the tumor response and carry out the meta-analysis, as they were independent groups. When analyzing the weight of the animals, the same animals were weighed at multiple time points throughout the study. The outcomes could only be evaluated independently at each time interval because the animals were grouped by different treatment periods, resulting in the same group being assessed more than once.

### 3.3 Meta-analysis – study outcomes

**Figure 3:**
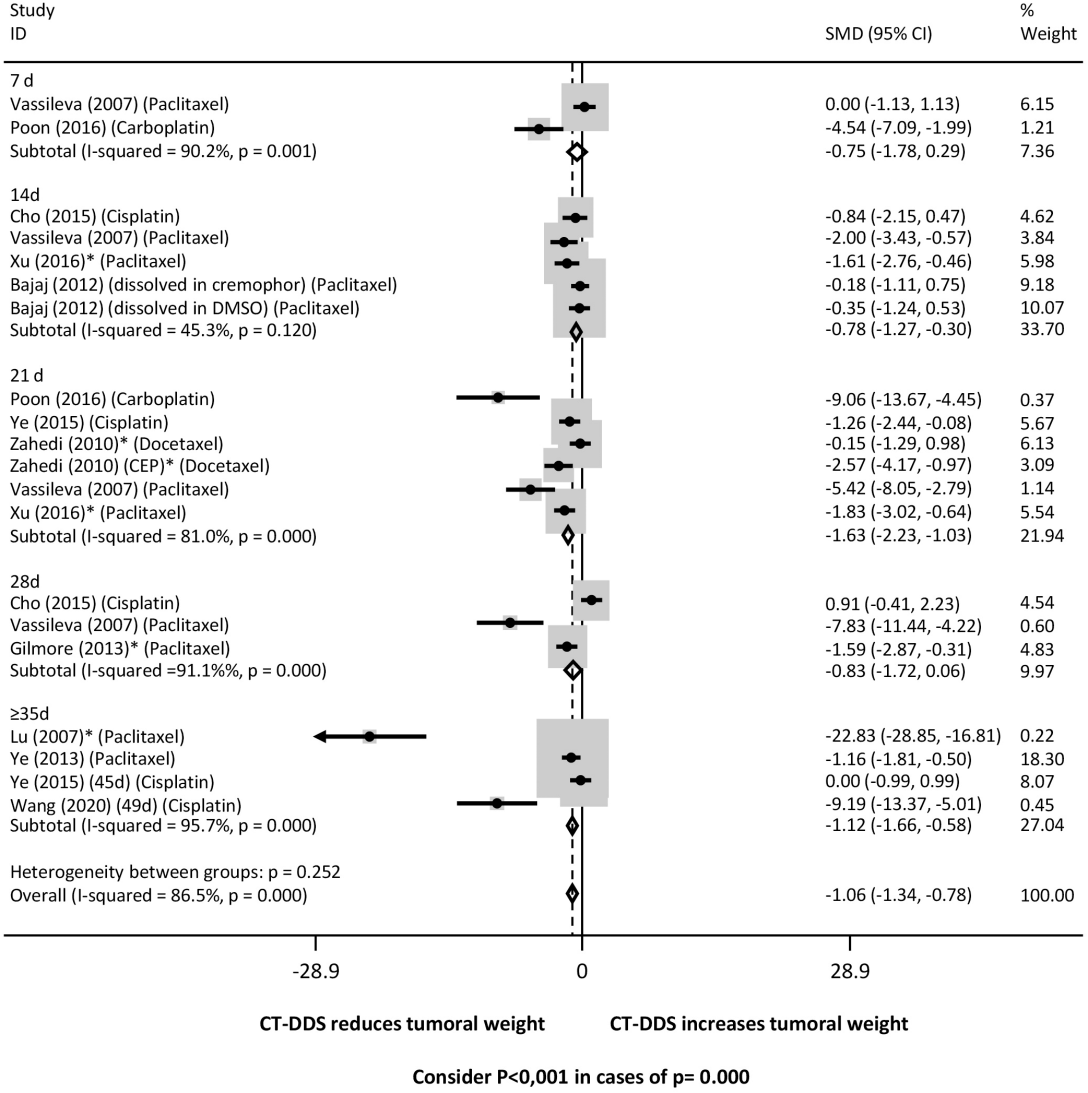
Tumor weight at different time points following intraperitoneal chemotherapy administered via drug delivery systems (DDS) compared to free intraperitoneal chemotherapy. Forrest plot presenting the pooled effect estimates from the meta-analysis with 95% confidence intervals (CIs) for each study. The size of each square represents the weight of each study in the meta-analysis, with larger squares corresponding to studies with greater weight. The horizontal lines through each square represent the confidence intervals for each study’s effect estimate. The diamond at the bottom of the plot represents the overall pooled effect estimate and its confidence interval. Heterogeneity across studies was assessed using the I² statistic, with values of 0-25%, 26-50%, 51-75%, and >75% indicating low, moderate, substantial, and high heterogeneity, respectively. A fixed-effect model was used for the analysis, assuming that all studies estimate the same underlying effect. * Studies that only evaluated xenografts other than SKOV3.

Tumor response was evaluated based on the tumor weight and tumor volume. Regarding the period of treatment (duration), tumor weight was lower in the group treated with DDS for: a) 14, 21 and 35 days and overall (grouping all time intervals) when compared with intraperitoneal free chemotherapy (**Figure 3**) and PBS (**Supplementary Figure 1**); b) 14, 21, and 35 days and overall, when compared with the empty device (**Supplementary Figure 2**). However, there was significant heterogeneity, varying from moderate to high, between groups.

**Figure 4:**
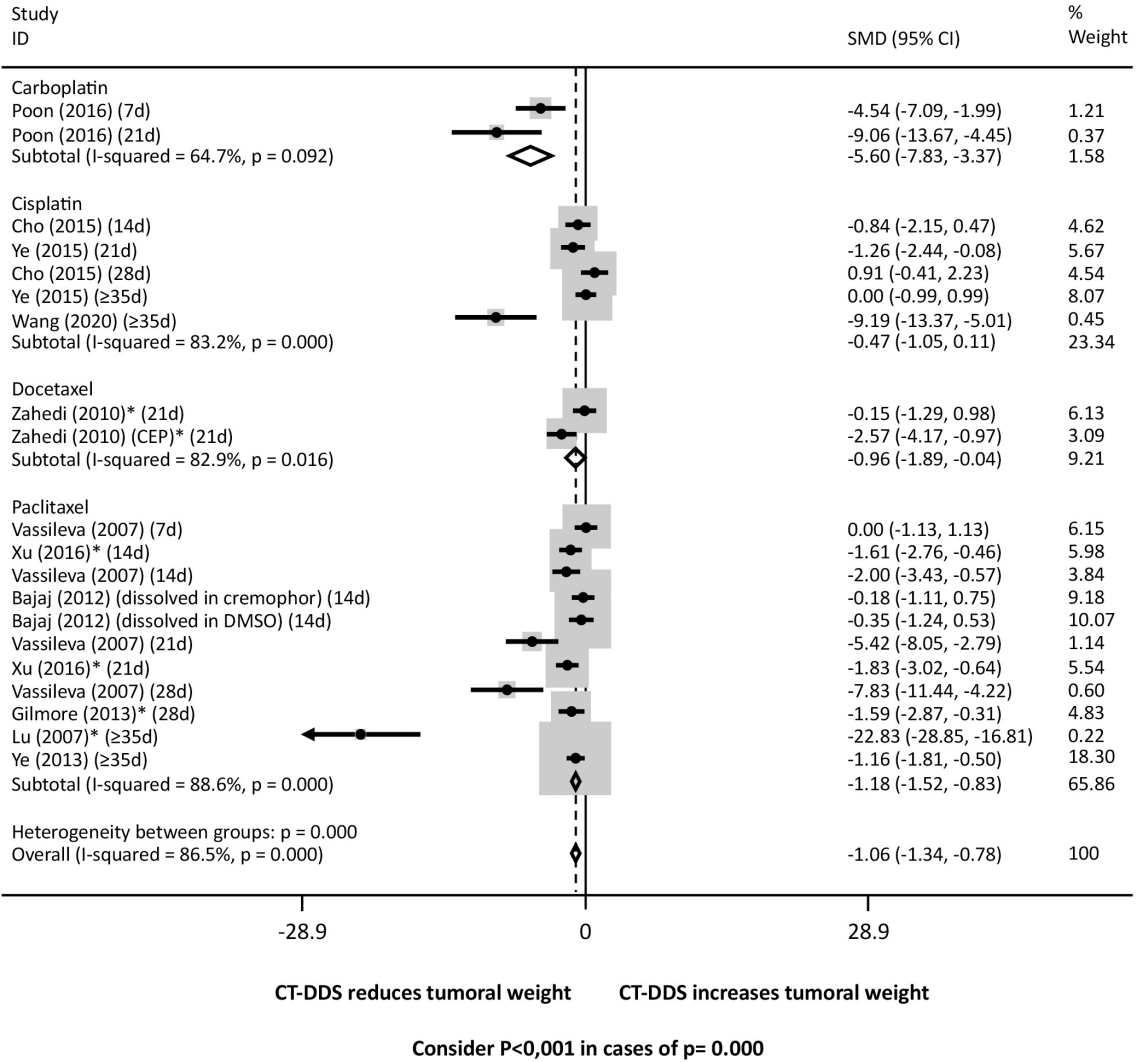
Tumor weight following intraperitoneal administration of various chemotherapy drugs via drug delivery systems (DDS) compared to free intraperitoneal chemotherapy. Consider P<0,001 in cases of p= 0.000. * Studies that only evaluated xenografts other than SKOV3.

Considering the chemotherapy drugs released by DDS, there was a significant regression in tumor weight in mice treated with DDS containing: a) carboplatin, docetaxel and paclitaxel and overall when compared with mice treated with intraperitoneal free chemotherapy (**Figure 4**); b) carboplatin, cisplatin, docetaxel, and paclitaxel compared with mice treated with PBS (**Supplementary Figure 3**); and c) docetaxel, paclitaxel and overall compared with mice treated with the empty device (**Supplementary Figure 4**). There was significant heterogeneity between all the three groups.

**Figure 5:**
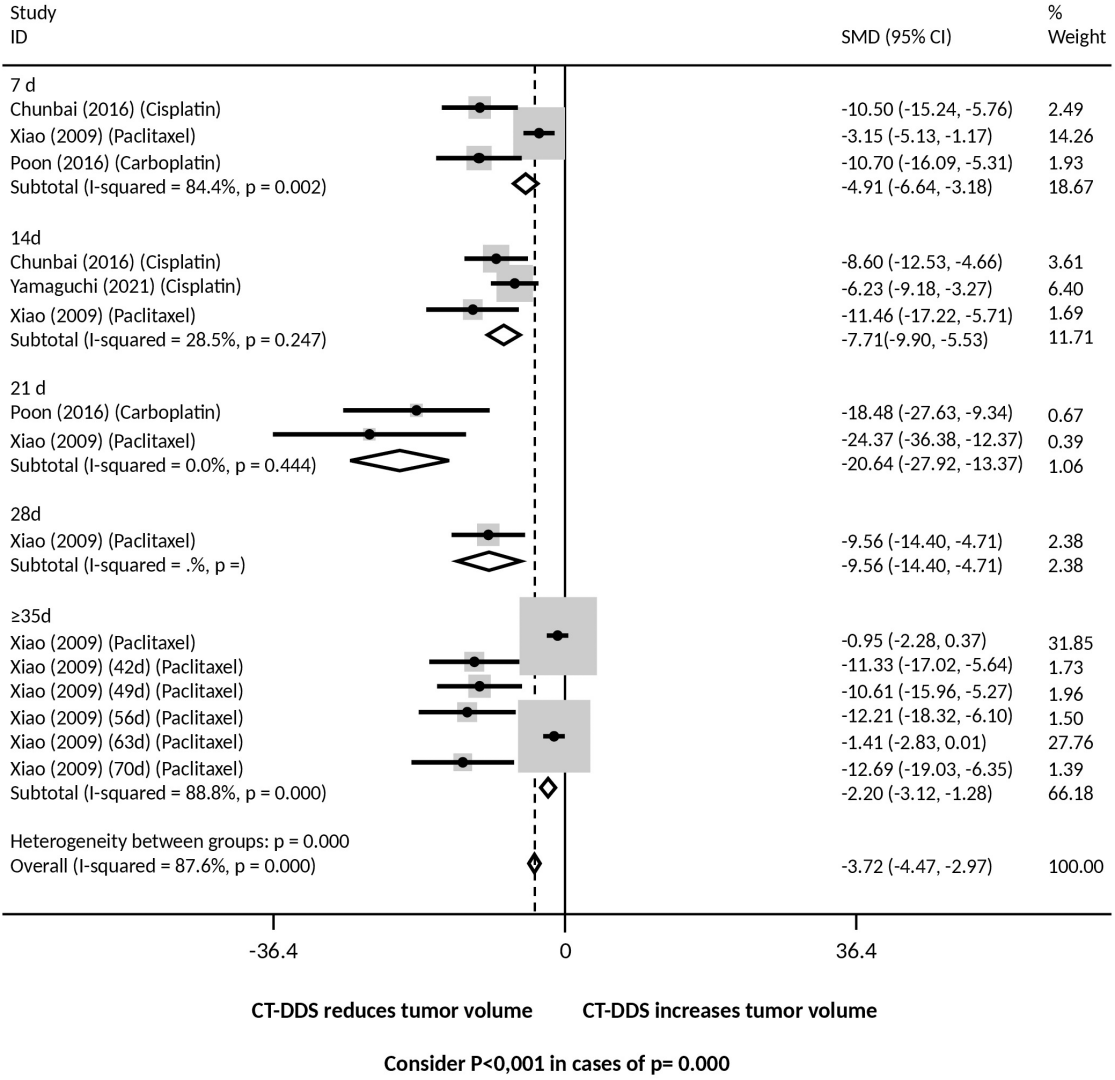
Tumor volume at different time points following intraperitoneal chemotherapy administered via drug delivery systems (DDS) compared to free intraperitoneal chemotherapy. Consider P<0,001 in cases of p= 0.000.

Tumor response was also evaluated based on the tumor volume. Regression of tumor volume was significantly greater in mice treated with DDS for all time periods (7 - ≥35 days) than in mice from all control groups, treated with intraperitoneal free chemotherapy (**Figure 5**), or PBS (**Supplementary Figure 5**), or the empty device (**Supplementary Figure 6**). There was significant heterogeneity between groups for all comparisons.

**Figure 6:**
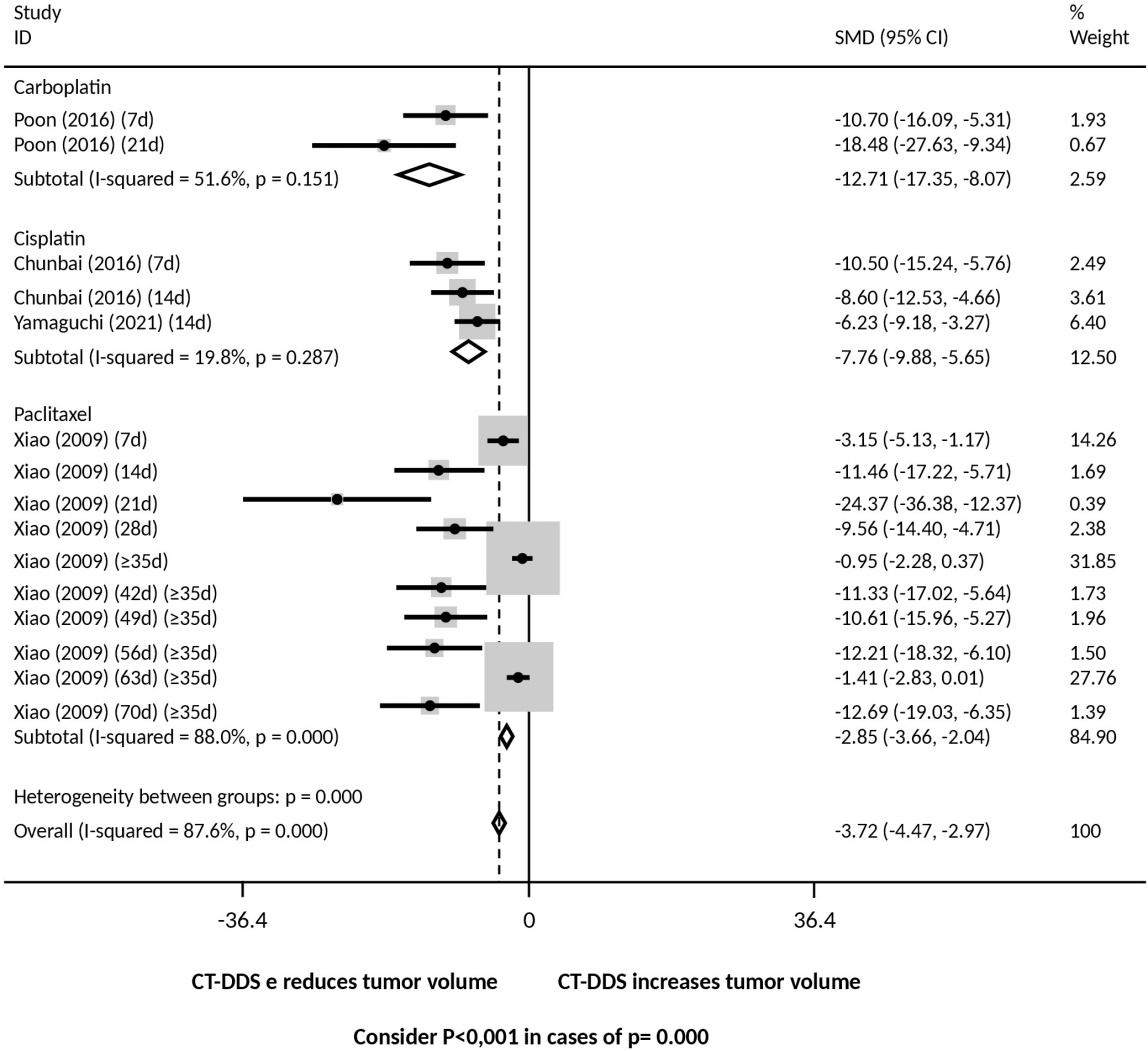
Tumor volume following intraperitoneal administration of various chemotherapy drugs via drug delivery systems (DDS) compared to free intraperitoneal chemotherapy. Consider P<0,001 in cases of p= 0.000.

Regarding chemotherapy drugs, there was a reduction in tumor volume for mice treated with DDS containing: a) carboplatin, cisplatin and paclitaxel, and overall, compared with mice treated with intraperitoneal free chemotherapy (**Figure 6**); b) all drugs tested, i.e., carboplatin, cisplatin, docetaxel, paclitaxel, and overall, compared with mice treated with PBS (**Supplementary Figure 7**); c) cisplatin and overall, compared with mice treated with the empty device (**Supplementary Figure 8**). Significant heterogeneity between groups was observed in comparisons of the DDS with PBS and intraperitoneal free chemotherapy.

We next evaluated animal body weight within treatments, because it was the most common evaluation of toxicity among studies. Indirect signs such as inactivity or change in eye color were not commonly described.

**Figure 7:**
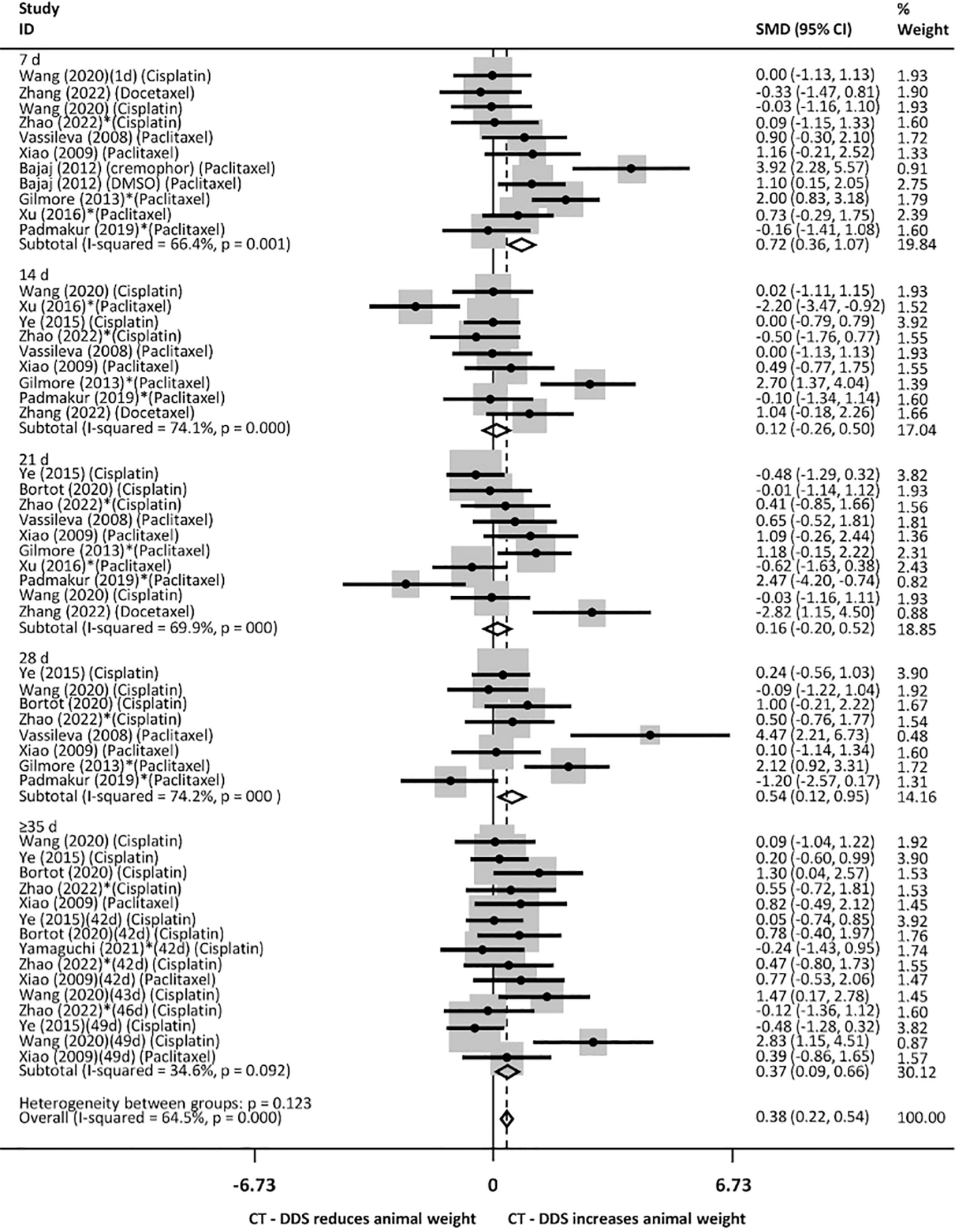
Animal weight at various time points following intraperitoneal administration of chemotherapy drugs via drug delivery systems (DDS), compared to free intraperitoneal chemotherapy. Consider P<0,001 in cases of p= 0.000. * Studies that only evaluated xenografts other than SKOV3.

Although 4/13 studies used percentage variation to evaluate weight gain or loss, these studies reported an initial mice weight of approximately 20g, which allowed us to estimate a numerical value and build the meta-analysis. Animals treated with DDS were heavier after 7, 28, and ≥35 days of treatment and overall, when compared to the group that received intraperitoneal free chemotherapy (**Figure 7**) for the same time period. There was no weight difference in mice treated with DDS in most time periods (7, 14, 21, 28 days), but mice treated for ≥35 days and overall were heavier when compared to mice that received PBS (**Supplementary Figure 9**). In addition, mice treated with DDS were less heavy at 4, 21 days and overall, but not at 7, 28 and ≥35 days, compared to animals treated with the empty device (**Supplementary Figure 10**). The studies that evaluated mice weight variation along the periods of treatment showed 64.5% of heterogeneity among them.

**Figure 8:**
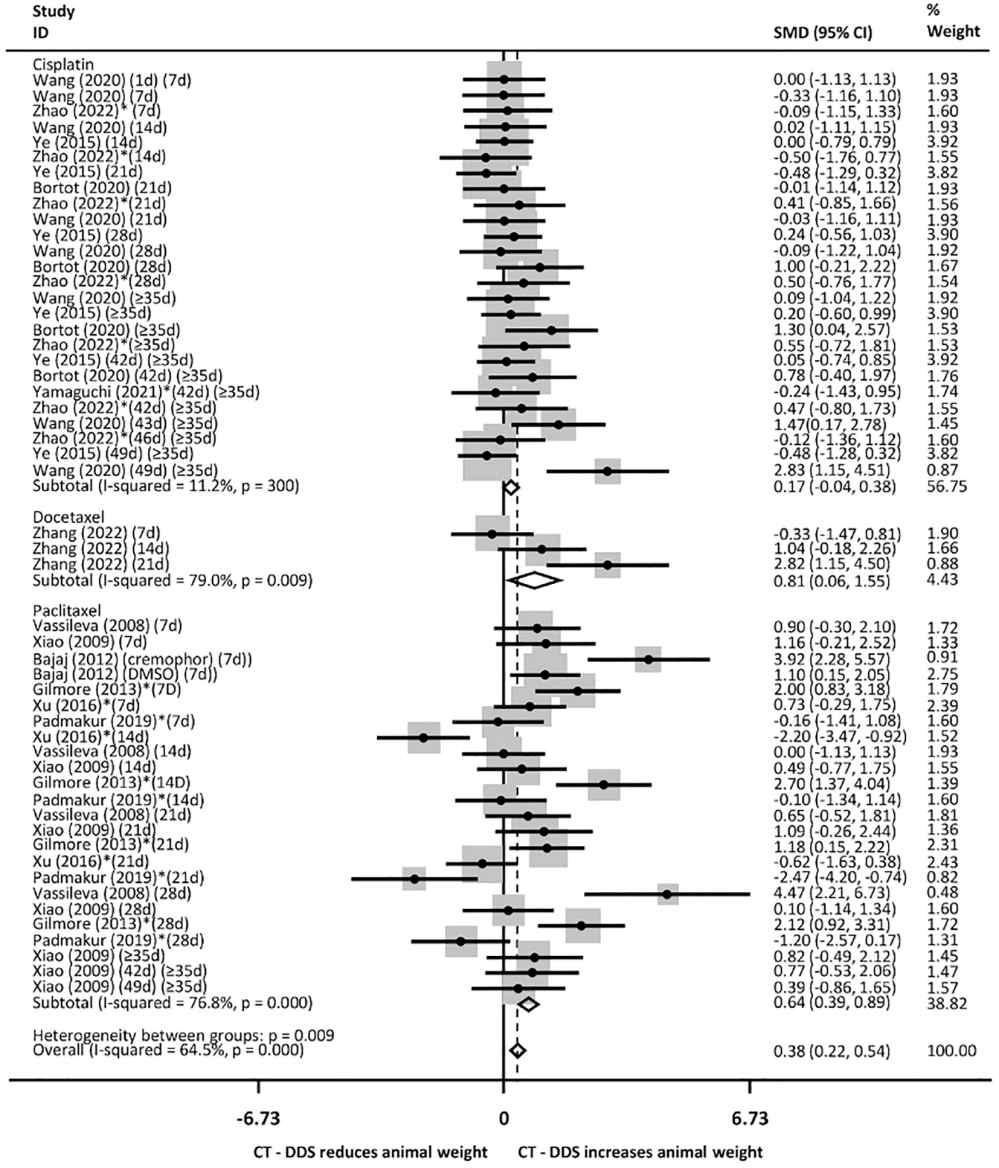
Animal weight following intraperitoneal administration of various chemotherapy drugs via drug delivery systems (DDS) compared to free intraperitoneal chemotherapy (over different time periods, from 7 days to more than 35 days). Consider P<0,001 in cases of p= 0.000. * Studies that only evaluated xenografts other than SKOV3.

Regarding the drugs used, mice treated with docetaxel, paclitaxel and overall were heavier, but not mice treated with cisplatin, when compared with mice treated with intraperitoneal free chemotherapy (**Figure 8**). There was no significant weight difference in mice treated with DDS containing docetaxel, and paclitaxel, but there was a reduced weight in mice treated with cisplatin and overall, when compared with the group treated with PBS (**Supplementary Figure 11**), with no heterogeneity between groups. Besides that, mice treated with DDS containing paclitaxel and overall were less heavy, but not those treated with DDS with cisplatin, than mice treated with the device devoid of chemotherapy (**Supplementary Figure 12**). There was heterogeneity between groups for comparisons of DDS with intraperitoneal free chemotherapy or the free device.

## 4 Discussion

We evaluated tumor response and treatment toxicity in studies that compared intraperitoneal devices of chemotherapy release with free chemotherapy intraperitoneal administration in animal models of ovarian cancer xenografts. In the meta-analysis we observed a small benefit in overall tumor reduction in animals treated with intraperitoneal chemotherapy applied through the slow release device compared with animals treated with intraperitoneal free chemotherapy. In addition, there was no important toxicity that negatively impacted animal weight in rodents treated with DDSs, compared with rodents treated with free chemotherapy.

Continuous, low-dose release of chemotherapy is called metronomic (37) and its efficacy is based mainly on compromising integrity of endothelial cells, reducing angiogenesis (33, 38). A long release time of the chemotherapy agent also allows the drug to achieve favorable distribution to different organs. and not just on the peritoneal surface (26, 38), and may have beneficial effects against inadequate distribution of intraperitoneal chemotherapy, that is a recurrent problem, possibly compromising peritoneal chemotherapy efficacy (39).

A previous meta-analysis, published in 2015, compared animal studies of drug delivery systems for ovarian cancer treatment (17). In this review, the outcomes were grouped into only one treatment time interval, which allowed a greater number of studies to be included in the same analysis. The selected studies included peritoneal and intravenous release formulations (17). In the present study, 15 new studies were available for inclusion, eight of which using cisplatin and seven using paclitaxel. In 2022, a publication of an equivalent systematic review on murine models of gastrointestinal cancer involving 35 studies showed an important clinical improvement in tumor reduction in animals that received the prototype, consisting of DDSs containing cytostatics for the treatment of gastro-intestinal peritoneal metastasis, compared to those that received free chemotherapy in the abdominal cavity (18). Mice survival was one important outcome in both studies (17, 18). In agreement some studies included in this meta-analysis showed an increased survival without important variation in animal weight. In most studies, animal survival was described in Kaplan-Meier curves, which made a meta-analysis not possible due to the absence of confidence intervals.

One of the major concerns with intraperitoneal delivery devices is erratic chemotherapy release that could eventually increase the toxicity of the treatment or interrupt the supply of medication prematurely. By separating the outcomes of each study into weekly intervals, we were able to unravel the benefit of DDSs in terms of the time after implantation. The maintained benefit over various time intervals of follow-up supports the advantage of the devices in favor of continued drug release, compared to free chemotherapy administered in bolus.

The present data show that slow drug release devices did not exhibit a deleterious impact on animal body weight compared with intraperitoneal free chemotherapy administration. Besides that, overall analysis of mice treated with DDS revealed they were less heavy than those treated without chemotherapy, i.e., PBS or the device devoid of the drug. Measurement of mouse weight as an outcome of treatment efficacy is controversial (40). Even though a decrease in body weight of > 20% is an important sign of toxicity and is prone to animal euthanasia, weight gain may be associated with tumor weight growth and not necessarily with adequate nutrition. In addition, weight stability is interpreted as an adequate indicator of favorable toxicity (37). No study in the present review described euthanasia associated with mouse weight reduction and weight stability is considered a favorable finding for treatment (37, 41).

An important finding described in most studies is tumor regression, evaluated by tumor weight, tumor volume or tumor bioluminescence. A meta-analysis of the tumor bioluminescence could not be performed because there was a significant variation in the way the studies expressed measurement units. The weight and volume of the residual tumor are direct and objective methods, however, in rodents, it is more reproducible in rats, because mice normally develop very small tumor implants, that are difficult to quantify (40).

Most studies evaluated less than ten mice in each group and, consequently, only a few reached statistical significance individually. In total, 11 studies compared the regression of tumor weight in the experimental groups, in comparison with PBS, and in nine of them the evaluation was undertaken after 21 days of treatment. Indeed, most devices are designed to slowly release medication over two weeks. Studies that evaluated continuous treatment after 21 days also report maintenance of good results after this period compared with free chemotherapy (36, 37, 42–44) and the device without medication (36, 44).

It is important to note that various types of devices that release chemotherapy drugs in different manners were evaluated in the studies, as is expected in investigational experiments (17, 18). The association of chemotherapy drugs with other agents that would assist in the intracellular transport of the substance is quite common and generally brings benefits in tumor regression. It is noteworthy that the most recent studies looked for presentations with nanoparticles and micelles because of their potential for greater penetration into neoplastic tissue (17).

The most commonly used chemotherapy drugs in the present review were paclitaxel and cisplatin, which are commonly used in clinical practice. There is great interest in the development of cisplatin slow release devices, because in a recent clinical trial, there was efficacy when was applied to the peritoneal cavity in the HIPEC procedure for interval ovarian cytoreduction (12).

In the present analysis, treatment of ovarian cancer xenografts with cisplatin intraperitoneal delivery devices elicited dubious benefits, i.e., enhanced or absent additional growth inhibition, as evaluated through tumor volume or tumor weight, respectively, compared with intraperitoneal free chemotherapy. A trend toward tumor reduction might be due to the optimization of cisplatin uptake by tumor cells through formulations with nanoparticles (45–47). In a previous systematic review, cisplatin intraperitoneal device also showed inconsistent results with no additional benefit compared to free drug administration regarding tumor inhibition, but with improved survival (17). The use of nanotechnology can assist in targeted release of hydrophobic agents, stabilization of transport molecules and reduction of systemic toxicity of antineoplastic agents (48).

In the present review, the performance of intraperitoneal paclitaxel release device was superior to that of intraperitoneal free chemotherapy. Paclitaxel has been used in most studies, mainly through nanoparticles. The contrast between the limited use of nanoparticles in chemotherapy formulations in clinical practice (49) and their high performance in *in vitro* studies (50) may have driven further research using murine models.

Few studies evaluated the performance of carboplatin delivered through intraperitoneal devices, but they showed favorable results in reducing both tumor weight and volume of ovarian cancer xenografts. Carboplatin has adequate intraperitoneal pharmacokinetics and has been used in HIPEC clinical studies in humans with good tolerability (51). In addition, carboplatin was used in the largest clinical trial for outpatient peritoneal chemotherapy (8), although it did not result in a survival gain, compared to conventional intravenous application in advanced ovarian cancer.

Docetaxel has a high cytotoxic potential *in vitro*, but it is not superior to most chemotherapy drugs in human studies (52) and its use is generally limited to relapsed cases of ovarian cancer. Three animal studies using a standardized formulation of docetaxel showed favorable results, but unfortunately, did not evaluate the same outcomes.

We used the SYRCLES instrument to identify biases. Animal studies do not usually detail some methodological steps, such as the random selection of animals and blinding of evaluators, which may compromise some aspects of the instrument, as previously reported (18).

This systematic review has some limitations. In most studies, animal survival was mainly shown in Kaplan-Meier curves, that precluded a meta-analysis due to the absence of confidence intervals. In addition, Xenografts bioluminescence could not be evaluated due to a lack of analysis standardization. We have then used tumor response as a surrogate of treatment benefit. However, tumor weight and volume are rather difficult to evaluate and lack precision in mice (40). In addition, a limited number of animals evaluated in each study and the large methodological variability led to marked heterogeneity regarding tumor weight and volume. The same scenario has been described in a previous study by Raave et al. (17). Furthermore, although most studies compared DDS to release chemotherapy intraperitoneally with the same chemotherapy administered intraperitoneally in bolus, the dose and regimen of administration varied and pharmacokinetic evaluations were performed in some, but not all of them. These differential chemotherapy doses and regimens may have also influenced heterogeneity between groups.

The major contribution of this meta-analysis is a comprehensive analysis of tumor response through the evaluation of tumor volume and tumor weight, considering the drugs employed and treatment duration, comparing the intraperitoneal drug delivery systems (DDSs) containing chemotherapy with intraperitoneal administration of free chemotherapy. In addition, treatment toxicity, through animal weight was evaluated considering the drugs used and time of treatment.

## 4 Conclusion

The present review further supports the notion that slow-release intraperitoneal chemotherapy devices are effective and safe in animal models of ovarian cancer.

## Data Availability

The original contributions presented in the study are included in the article/**Supplementary Material**. Further inquiries can be directed to the corresponding author.
