# Supplementary material for: Peritoneal chemotherapy delivery systems for ovarian cancer treatment: systematic review of animal models
Source: Front Oncol. 2025 Jan 8;14:1487376. doi: 10.3389/fonc.2024.1487376 (PMC11750819; doi:10.3389/fonc.2024.1487376)
Supplement: Supplementary file 1 [file DataSheet1.docx]

Supplementary Table 1.Search Strategy

| Population | Intervention | Comparison | Outcomes |
| --- | --- | --- | --- |
| "Ovarian" OR "carcinomatosis" | ("Polymers"[Mesh] OR "Drug Delivery Systems"[Mesh] OR "Absorbable Implants"[Mesh] OR "Phospholipids"[Mesh] OR "Delayed-Action Preparations"[Mesh] OR "Infusion Pumps, Implantable"[Mesh] OR "Chitosan"[Mesh] OR "Polyvinyl Alcohol"[Mesh] OR "sustained release" OR "slow release" OR "controlled release" OR "membrane" OR "hydrogel" OR "Polyethylene Glycol Acid" OR "Implant System" OR "Injectable Biomaterial" OR "Continuous Release" OR "continuous intraperitoneal delivery" OR "continuous chemotherapy" OR "continuous docetaxel" OR "continuous cisplatin” OR "continuous paclitaxel” OR "continuous carboplatin” OR “micellar” OR “micelle" | "Intraperitoneal" OR "peritoneal" | "Toxicity" OR "survival" OR "treatment" OR "tumor burden" |

Supplementary figures

fig S1: *Forest Plot* Weekly Tumor Weight: DDS *vs*. PBS


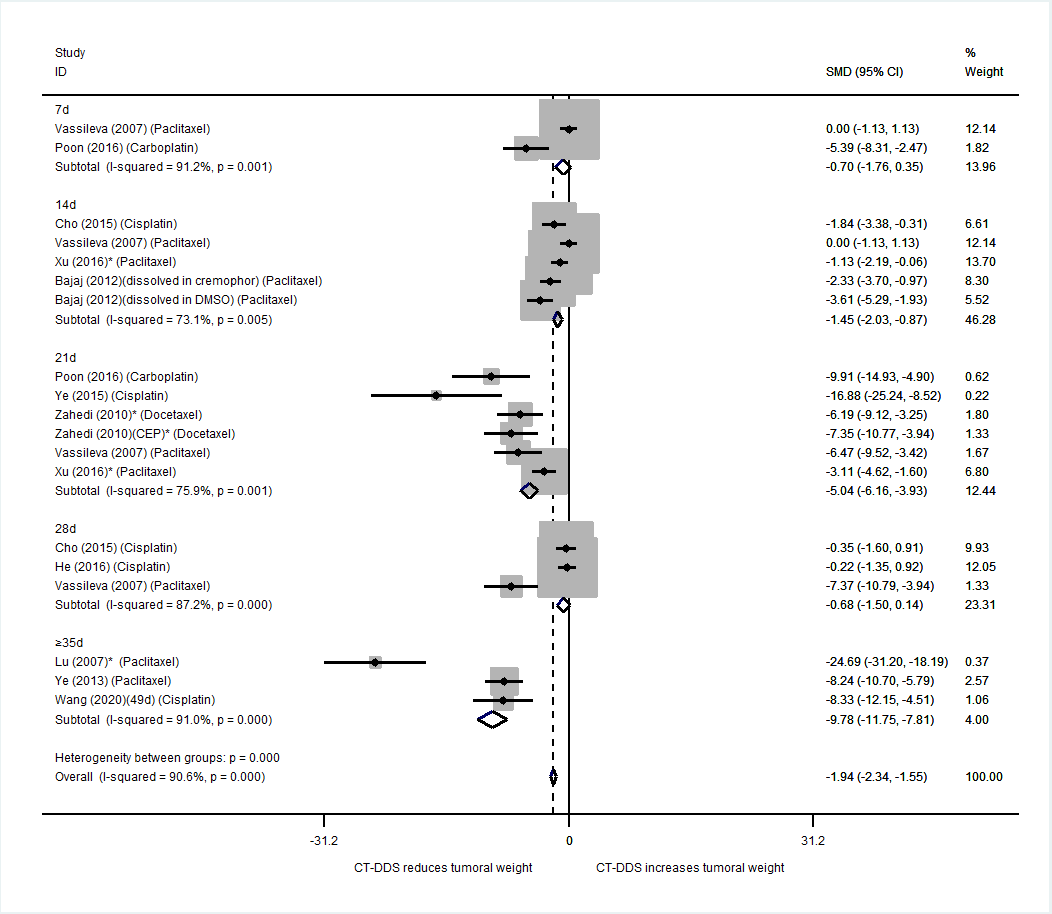


Consider P<0,001 in cases of p= 0.000

fig S2: *Forest Plot* Weekly Tumor Weight: DDS *vs*. empty device


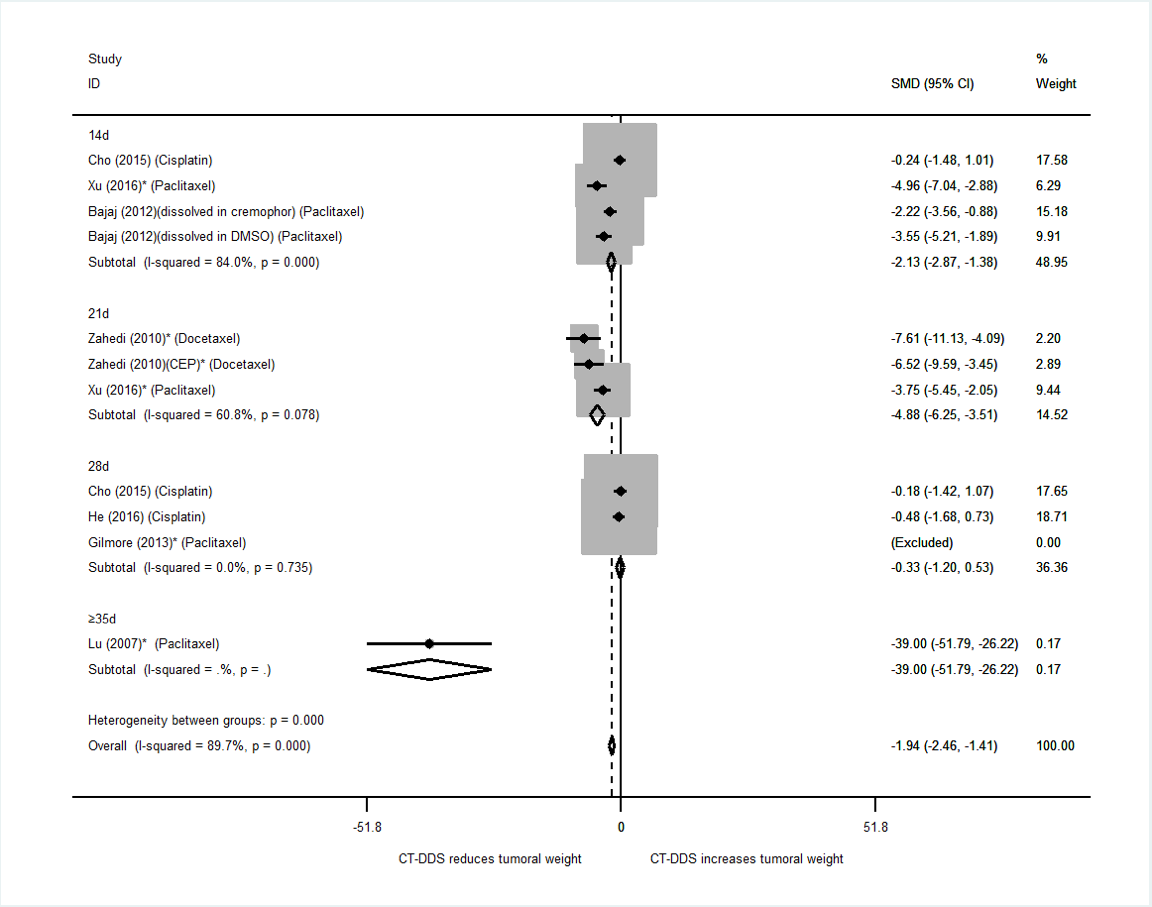


Consider P<0,001 in cases of p= 0.000

Figure S3: *Forest Plot* Tumor Weight According to Each Drug: DDS vs PBS


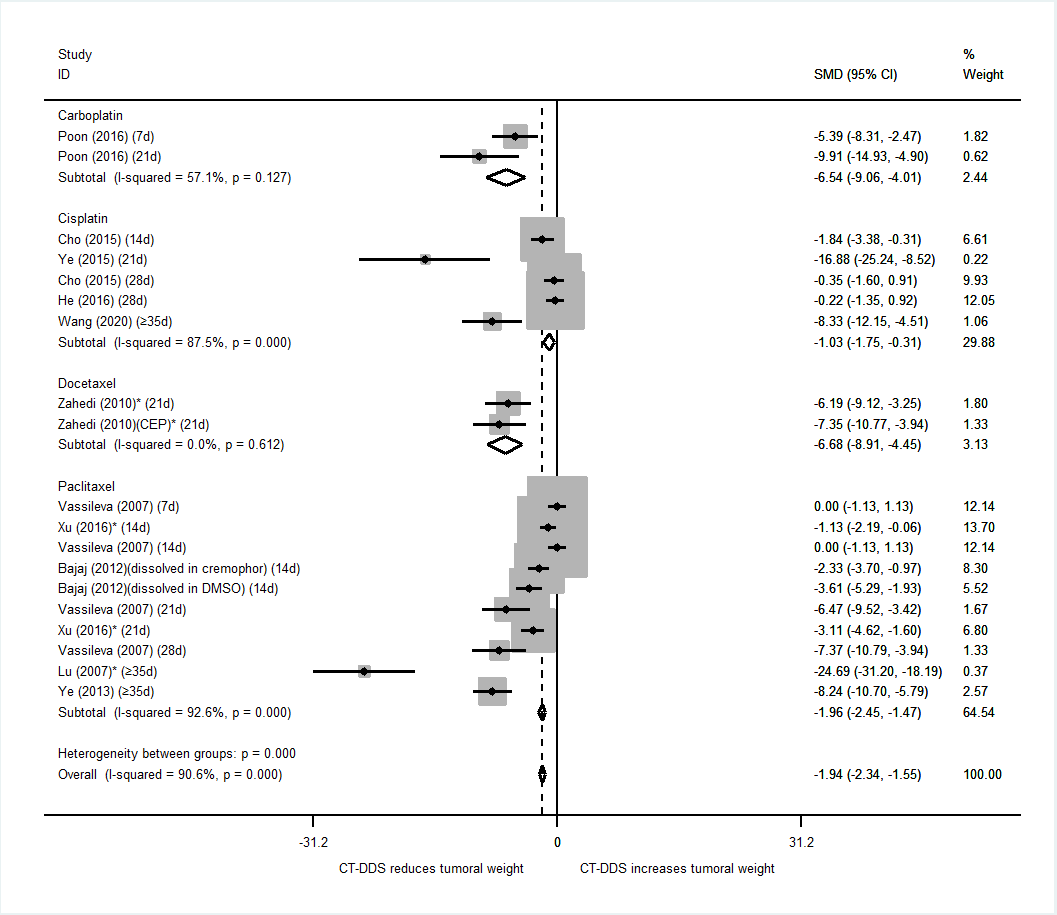


Figure S4: *Forest Plot* Tumor Weight According to Each Drug: DDS vs empty device


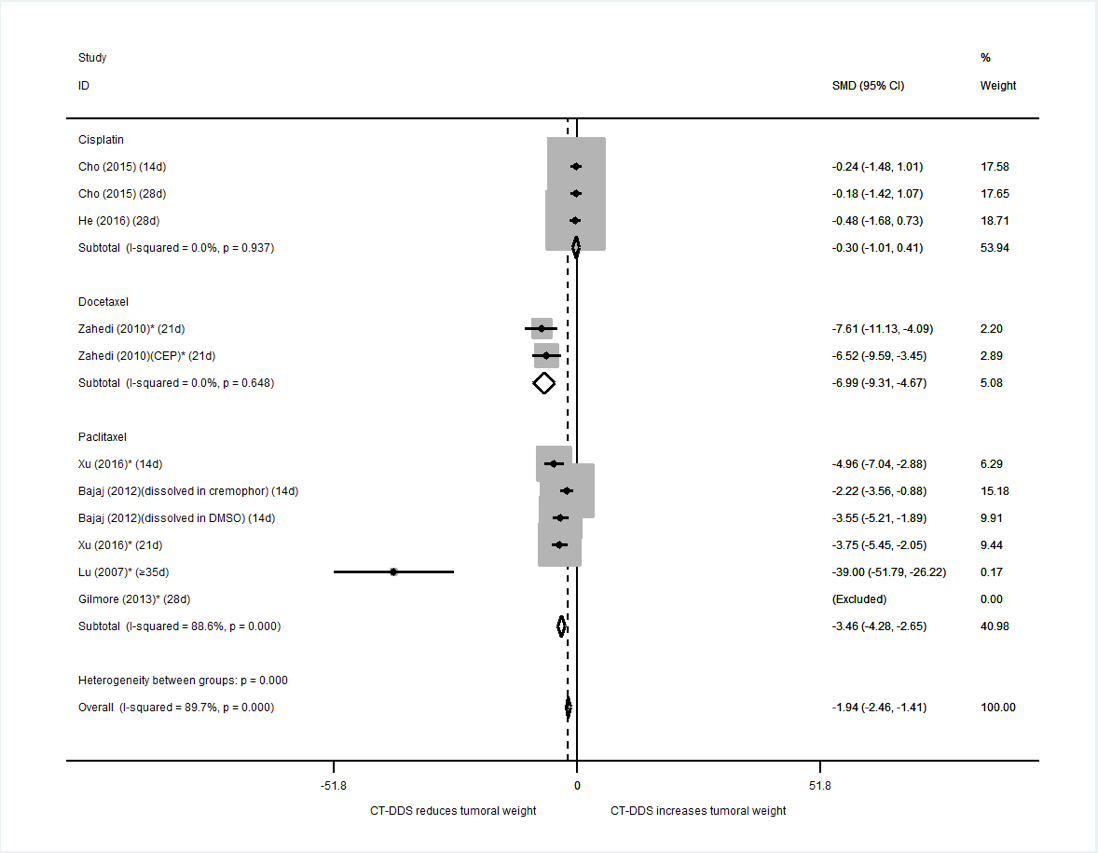


Consider P<0,001 in cases of p= 0.000

Figure S5 *Forest Plot* Weekly Tumor Volume: DDS *vs*. PBS


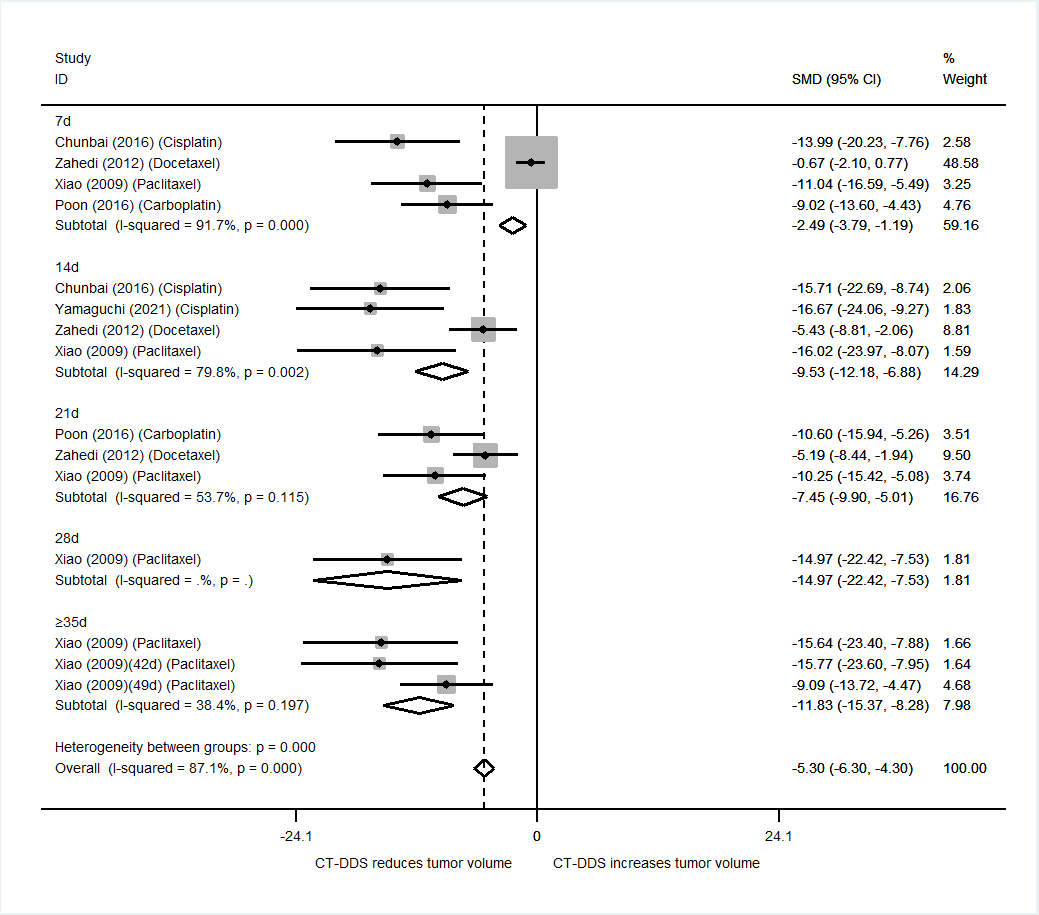


Consider P<0,001 in cases of p= 0.000

Figure S6 *Forest Plot* Weekly Tumor Volume: DDS *vs*. empty device


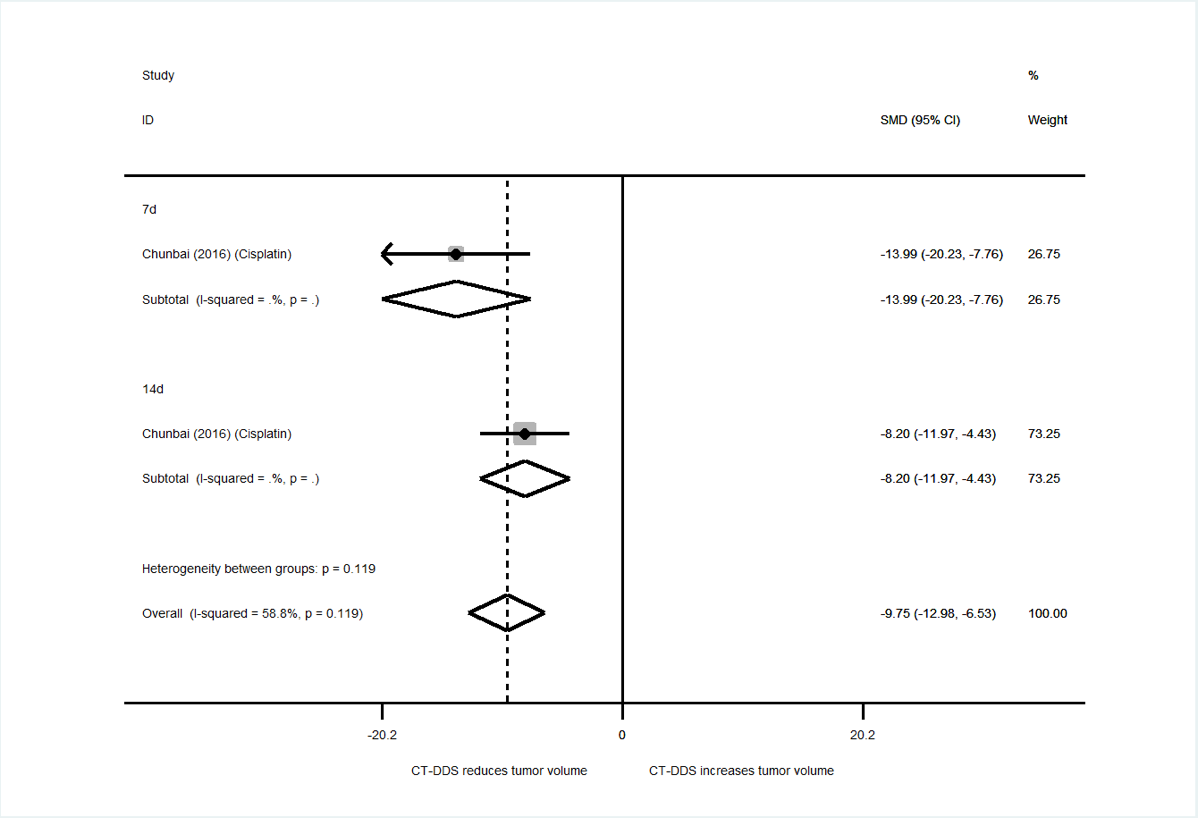


Figure S7 *Forest Plot* Tumor Volume According to Each Drug: DDS vs. PBS


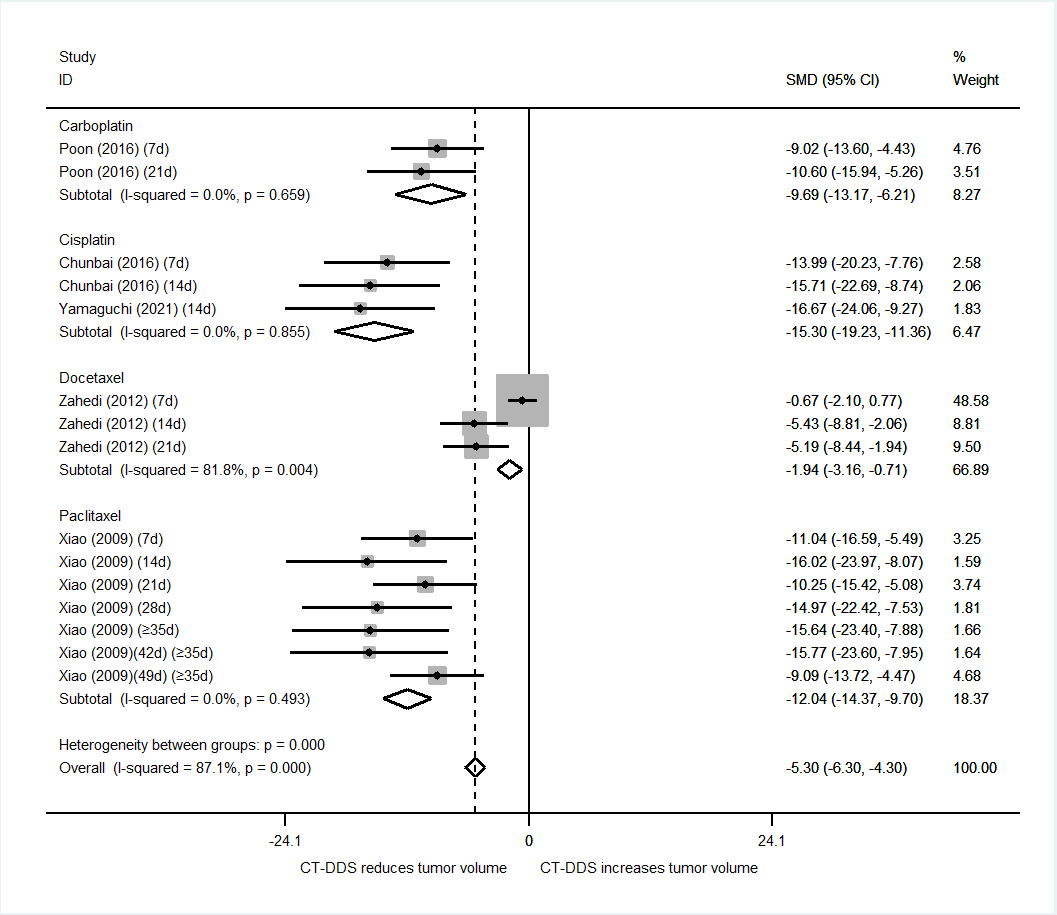


Consider P<0,001 in cases of p= 0.000

Figure S8 *Forest Plot* Tumor Volume According to Each Drug: DDS vs. empty device


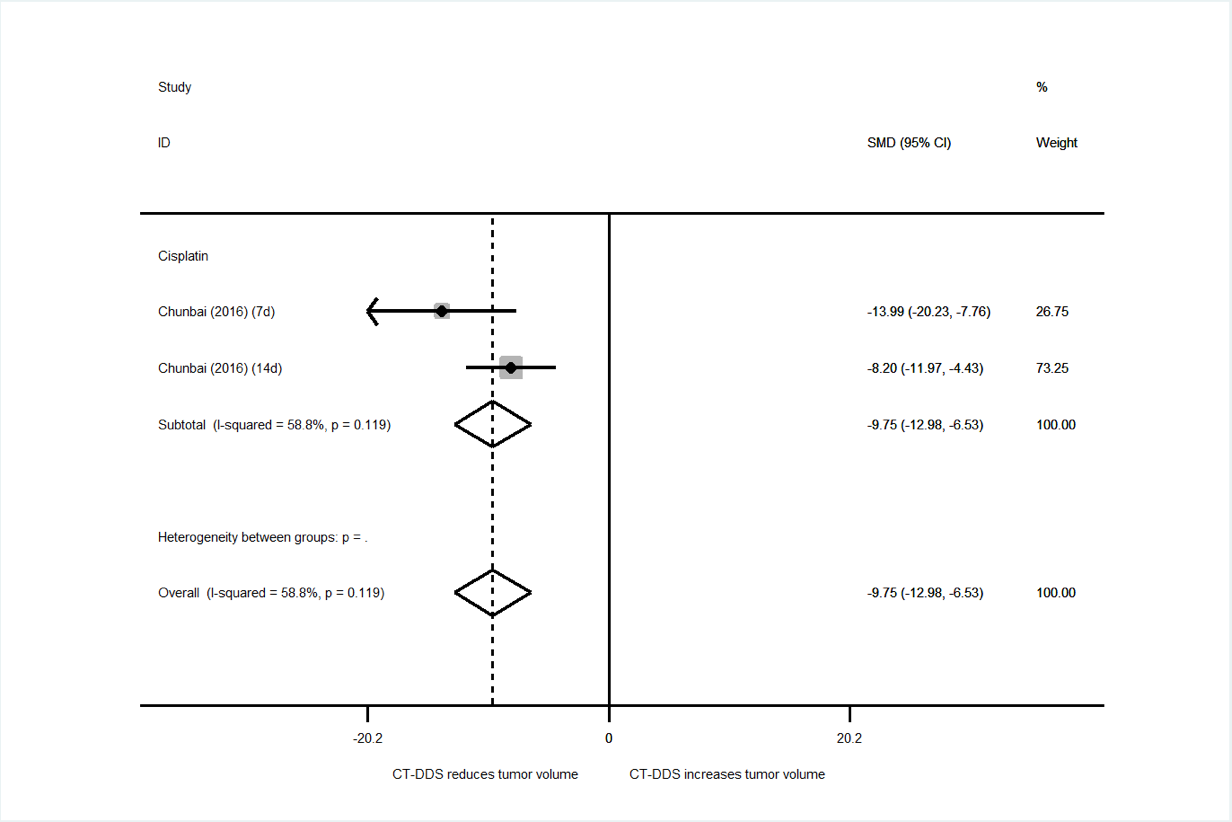


Figure S9- F*orest Plot* Weekly Mice Weight: DDS *vs*. PBS


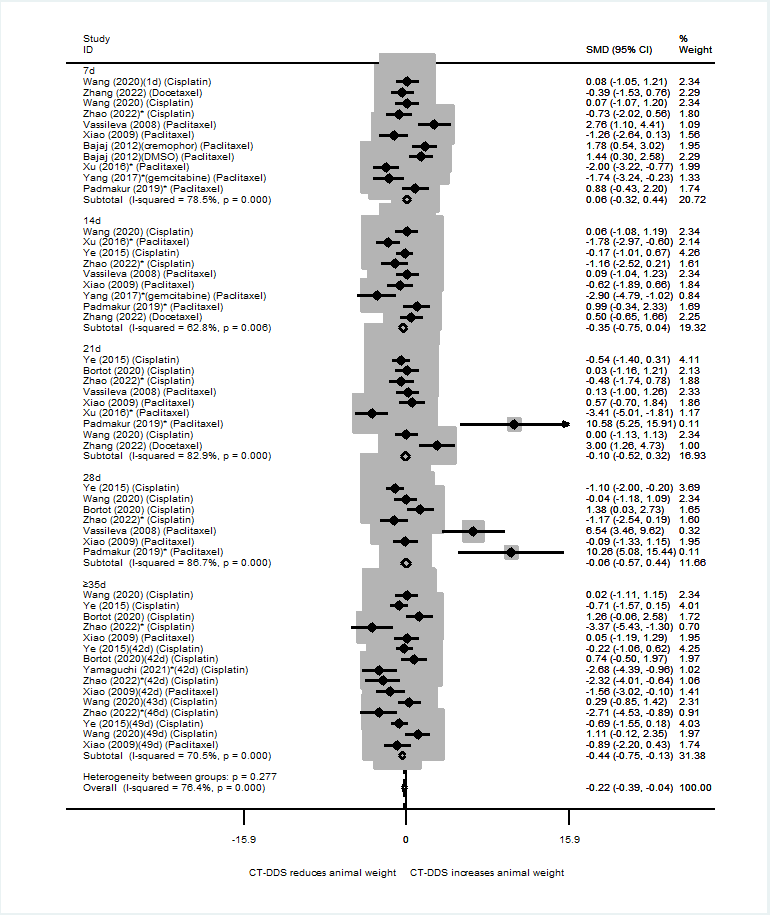


Consider P<0,001 in cases of p= 0.000

Figure S10 F*orest Plot* Weekly Mice Weight: DDS *vs*. empty device


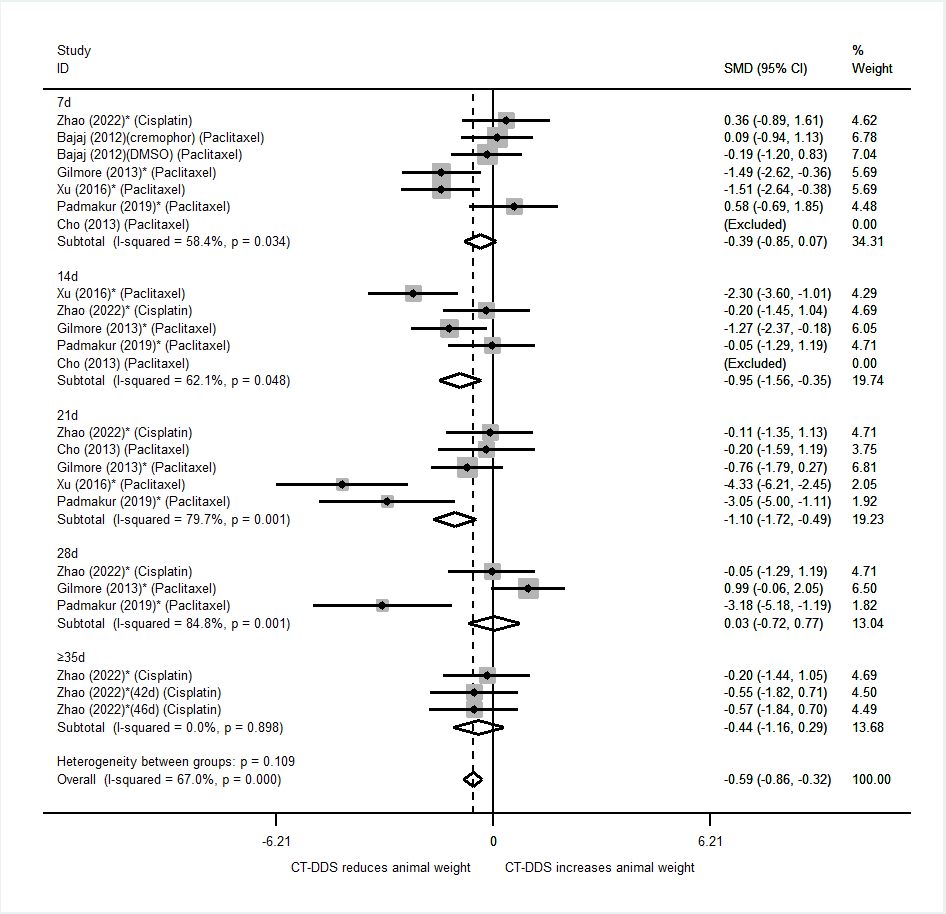


Consider P<0,001 in cases of p= 0.000

Figure S11 – *Forest Plot* Mice Weight According to Each Drug: DDS *vs*. PBS


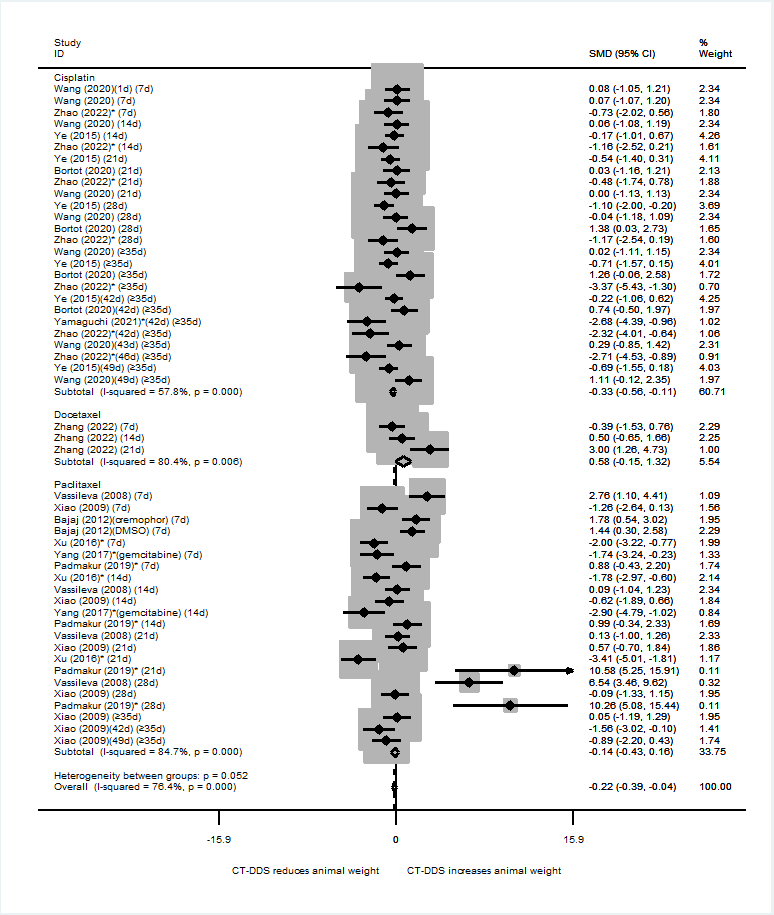


Consider P<0,001 in cases of p= 0.000

Figure S12 – *Forest Plot* Mice Weight According to Each Drug: DDS *vs*. empty device


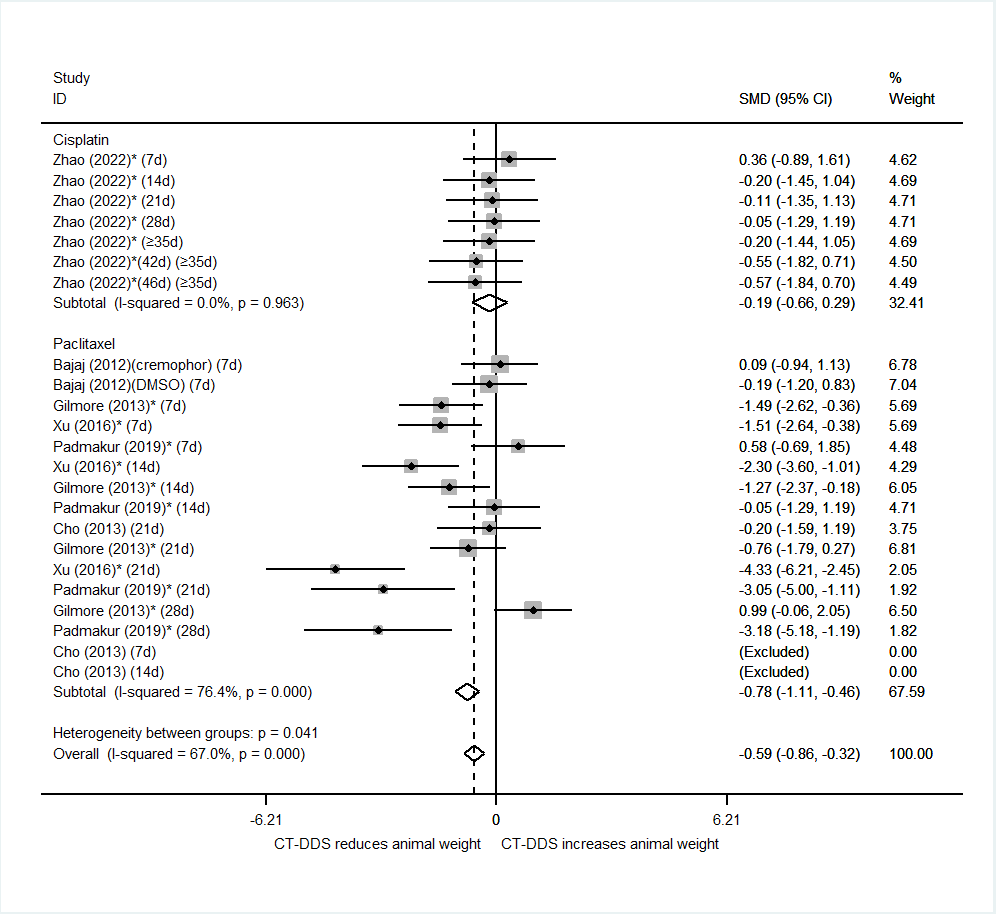


Consider P<0,001 in cases of p= 0.000
